# Insights into antibiotic resistance promoted by quinolone exposure

**DOI:** 10.1128/aac.00997-24

**Published:** 2024-11-26

**Authors:** Natassja G. Bush, Isabel Diez-Santos, Pilla Sankara Krishna, Bernardo Clavijo, Anthony Maxwell

**Affiliations:** 1 Department of Biological Chemistry, John Innes Centre, Norwich Research Park 443466 , Norwich, United Kingdom; 2 School of Biological Sciences, University of East Anglia School of Biological Sciences 98950 , Norwich, United Kingdom; 3 Earlham Institute, Norwich Research Park 150238 , Norwich, United Kingdom; 4 Department of Molecular Microbiology, John Innes Centre, Norwich Research Park 534072 , Norwich, United Kingdom; Bill & Melinda Gates Medical Research Institute, Cambridge, Massachusetts, USA

**Keywords:** topoisomerase, gyrase, fluoroquinolones, antibiotic resistance

## Abstract

Quinolone-induced antibiotic resistance (QIAR) refers to the phenomenon by which bacteria exposed to sublethal levels of quinolones acquire resistance to non-quinolone antibiotics. We have explored this in *Escherichia coli* MG1655 using a variety of compounds and bacteria carrying a quinolone-resistance mutation in gyrase, mutations affecting the SOS response, and mutations in error-prone polymerases. The nature of the antibiotic-resistance mutations was determined by whole-genome sequencing. Exposure to low levels of most quinolones tested led to mutations conferring resistance to chloramphenicol, ampicillin, kanamycin, and tetracycline. The mutations included point mutations and deletions and could mostly be correlated with the resistance phenotype. QIAR depended upon DNA gyrase and involved the SOS response but was not dependent on error-prone polymerases. Only moxifloxacin, among the quinolones tested, did not display a significant QIAR effect. We speculate that the lack of QIAR with moxifloxacin may be attributable to it acting via a different mechanism. In addition to the concerns about antimicrobial resistance to quinolones and other compounds, QIAR presents an additional challenge in relation to the usage of quinolone antibacterials.

## INTRODUCTION

Antimicrobial resistance (AMR) is a global threat with resistance to all classes of antibiotics reported worldwide. It is estimated that if nothing is done to mitigate this threat, by 2050 AMR will cause 10 million deaths per year and cost the world economy 10 trillion USD in economic output (1). This issue is compounded by a lack of innovation and few new structural classes of antibiotics being brought to the clinic (1–6). The WHO and O’Neill reports have both identified the importance of antibiotic stewardship and antibiotic R&D in tackling antibiotic resistance (1, 7). In addition to this, a better understanding of resistance mechanisms and bacterial evolution under the selection pressure of antibiotics is needed. Many complex biochemical and physiological processes are involved in the development of resistance, and there is often a lack of fundamental understanding of these processes (8, 9).

One complexity is the effect that sublethal antibiotic doses have on resistance development and selection. A number of studies and reviews (9–13) describe and discuss the mutagenic effect of low concentrations of antibiotics on bacteria, suggesting that sublethal doses of antibiotics can select for resistance by promoting homologous recombination (14) and horizontal gene transfer (15, 16), by induction of the SOS response (17, 18), or by RpoS induction (19). Exposure to sublethal concentrations of antimicrobials has also been shown to increase the number of persister cells (20–24), as well as the enrichment of resistance genes within the population (11). Bacteria can be subjected to subinhibitory levels of antibiotics in a variety of environments. These include concentration gradients due to spatial variation within the human body (25), excretion of unabsorbed antibiotics into the natural environment (26–29), and non-medical uses of antibiotics, such as agricultural use (12, 30) or through effluent from pharmaceutical plants (10, 29).

Quinolones are a potent class of synthetic antibacterials that target DNA gyrase and DNA topoisomerase IV (topo IV) (31). Fluoroquinolones (FQs) are the most successful class of bacterial topoisomerase antibacterials and are widely prescribed in the USA and Europe (32, 33). This, however, has led to an increase in resistance, including upregulation of efflux pumps, plasmid-based resistance, or mutations in the gyrase or topo IV genes (34, 35). This widespread resistance has resulted in revised stewardship for quinolones (33) as well as the WHO categorizing FQs as “critically important antimicrobials” (36).

Although the rise in FQ resistance is well-documented, there is also evidence that exposure to subinhibitory levels of quinolones can lead to resistance to non-quinolone antibiotics including multidrug resistance. Studies on antibiotic resistance found in farm animals treated with quinolones have found multidrug resistance. For example, 77% of *Escherichia coli* isolates from pre-weaned dairy calves treated with enrofloxacin for diarrhea and respiratory diseases showed resistance to three or more antimicrobials (37). A study on the effect of enrofloxacin on commensal *E. coli* populations in healthy chickens found that there was an increase in the number of isolates resistant to doxycycline and amoxicillin as well as to enrofloxacin (38). This was mirrored by studies looking at the effect of enrofloxacin on *E. coli* from chickens and turkeys. *E. coli* from the chickens was multidrug resistant after treatment (39), while the isolates from turkeys were resistant to β-lactams despite a lack of β-lactam exposure (40). However, these studies did not analyze the resistance mechanisms, so it is possible that the results do not reflect quinolone-induced resistance described here.

Treating bacterial cultures with quinolones directly has shown a similar result. One study showed an increase in the resistance of quinolone-susceptible methicillin-resistant *Staphylococcus aureus* to nafcillin when exposed to subinhibitory doses of ciprofloxacin (CIP) or levofloxacin (41). Another demonstrated that treatment of *E. coli* MG1655 with norfloxacin (NOR) led to increased resistance to kanamycin (KAN) and ampicillin (AMP), with the authors suggesting that the mechanism behind this resistance was due to an increase in mutagenesis as a result of oxidative stress caused by the quinolone (42).

Other work has shown that treatment of bacterial cultures with sublethal levels of quinolones increases mutation rates, mutation frequencies, and recombination (14, 15, 43–46). Some of these studies found single-point mutations in specific genes that would confer resistance to rifampin (RIF) or trimethoprim (TMP), further indicating the potential of quinolone-induced antibiotic resistance (QIAR). The cause of the increase in mutation rates has been linked to the induction of the SOS response (the main bacterial response to DNA damage [47]) and concomitant derepression of the error-prone polymerases (18, 48, 49). When there is DNA damage, the recombinase RecA stimulates the autocleavage of LexA leading to derepression of the SOS operon. This activates transcription of several genes, like the error-prone polymerases *dinB*, *polB*, and *umuDC*, which are “prone” to make mutations when repairing DNA. Quinolones have been shown to activate the SOS response, presumably by the generation and release of DNA breaks (50–52). As a result of this and the assumption that induction of SOS includes activation of the transcription of error-prone polymerases, several groups have investigated whether quinolone-induced mutations are caused by these polymerases. However, while some of these mutations have been linked to the SOS-activated error-prone polymerases (13, 53), others have been shown to be independent of the SOS response (54).

While there is evidence for QIAR and for the ability of quinolones to affect mutation rates, how different quinolones lead to QIAR and what mutations are associated with this induced resistance are not well understood. There are potentially important implications associated with these findings, as exposure of bacteria to subinhibitory levels of quinolones could have significant effects on general antibiotic resistance in the community. Using *E. coli* as a model, we sought to address the question of how and what kind of resistance is induced by sublethal treatment with quinolones, as well as what role the SOS response may play in the induction of QIAR.

## MATERIALS AND METHODS

### Bacterial strains and growth medium

*E. coli* strains, including the wild-type strain MG1655 (RefSeq accession no. NC_000913.3), were grown in Luria-Bertani (LB) medium (10 g/L NaCl, Sigma, 10 g/L tryptone, Oxoid, and 5 g/L yeast extract, Oxoid) supplemented with 1.5% (wt/wt) agar (Formedium) when indicated. (For a complete list of *E. coli* strains and the plasmids and primers used, please see the supplemental material.)

### Antibiotic susceptibility tests

Minimum inhibitory concentration (MIC) values were determined in triplicate as described (55). The antibiotics and antibacterials tested were the quinolones CIP, moxifloxacin (MXF), oxolinic acid (OXO), NOR, the aminocoumarin coumermycin A1 (COU), the aminoglycosides KAN, streptomycin (STR), the penicillin compound AMP, the phenicol compound chloramphenicol (CHL), tetracycline (TET), RIF, triclosan (TRI), the dihydrofolate reductase inhibitor TMP, and the antineoplastic agent mitomycin C (MMC). All compounds were purchased from Sigma.

### Antibacterial testing

**Fig 1:**
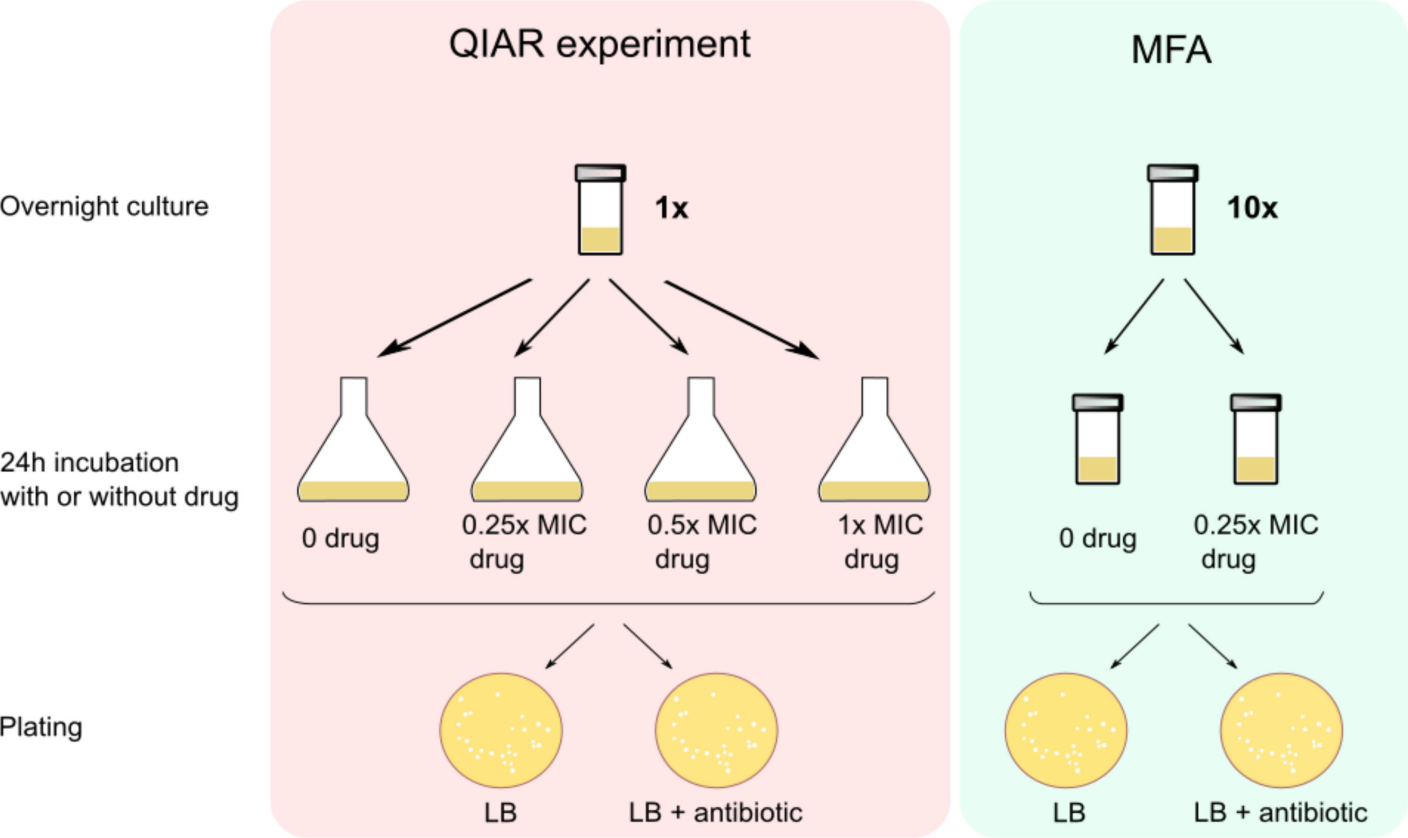
Scheme illustrating the protocols for QIAR experiments and mutant frequency assays (MFAs) following treatments with a quinolone or other compounds.

To measure QIAR, a colony from an *E. coli* strain was inoculated into 5 mL of LB and incubated overnight at 37°C (Fig. 1A). Fifty microliter of the overnight culture was inoculated into 50 mL of LB with either 0, 0.25×, 0.5×, or 1× MIC of a compound. If DMSO was the solvent that the compound was dissolved into, and then it was added to all samples at the same percentage. The samples were incubated at 37°C for 24 h. After incubation, 12.5 mL of the cultures was decanted into 50 mL centrifuge tubes and centrifuged for 30 min at 3,000 g. The pellet was resuspended in 2.5 mL LB. Serial dilutions were made from this prepared culture and plated on 9 cm LB 1.5% agar plates. Four hundred microliter from the remaining resuspension was plated on 15 cm LB 1.5% agar plates supplemented with either 50 µg/mL AMP, 50 µg/mL KAN, 30 µg/mL CHL, 12 µg/mL TET, 0.35 µg/mL CIP, 40 µg/mL COU, 8 µg/mL OXO, 0.64 µg/mL MXF, 0.064 µg/mL NOR, or 40 µg/mL MMC. Plates were incubated for 24 h at 37°C and then for 24 h at room temperature. To confirm antibiotic resistance, colonies that grew on the selection plates were re-streaked onto the antibiotic that they were challenged with and grown for 24 h at 37°C. To calculate resistance frequencies, the number of colonies that survived the re-streaking step was divided by a total number of colonies plated.

Additionally, a similar method was employed for mutant frequency assays MFAs (Fig. 1B), where *E. coli* colonies were grown overnight, split into two tubes, and exposed to no drug or 0.25× MIC of CIP or MMC. After 24 h of incubation, cultures were centrifuged, resuspended, and plated for colony-forming unit determination. The cultures were also plated on agar with eight times the MIC of CHL to identify CHL-resistant mutants. The frequency of CHL-resistant mutants was calculated as in the QIAR assay.

### Multidrug resistance assay

To assess multidrug resistance, CHL-resistant colonies obtained from a QIAR assay, in which the cells were treated with CIP, were cultured in LB medium for 24 h without antibiotic selection. These cultures were then streaked onto plates containing 2× the MIC of the following antibiotics: CHL, AMP, CIP, KAN, RIF, STR, TET, TRI, and TMP. Colonies that grew on the antibiotic-containing plates after three independent replicates were considered resistant to the corresponding antibiotic.

### Topoisomerase assays

Enzymes and DNA were obtained from Inspiralis Ltd (Gyrase: G1001; topo IV: T4001; plasmid pBR322: S5001 and R5001). *E. coli* gyrase and topo IV assays and IC_50_ and CC_50_ determinations were carried out according to the manufacturer’s protocols.

### Analysis of whole-genome sequences

*E. coli* strains were sent for whole-genome sequencing to MicrobesNG (http://www.microbesng.uk). Short-read sequencing was done on either Illumina MiSeq or Hiseq 2500 platforms. Trimmed reads were assembled against the *E. coli* MG1655 reference genome (NCBI—Accession: NC_000913.3). Assemblies and variant calling were performed using Snippy v3.1 (https://github.com/tseemann/snippy) with Perl v5.16.2, BioPerl v1.6.923, SamTools v1.3.1, bwa-mem v0.7.5, and Java v7.21. Assemblies and alignments were visualized using Tablet (v1.17.08.17) (56) to identify deletions and amplifications. All information about the various genes identified with mutations was obtained from EcoCyc database online (57). The trimmed reads can be found in Zenodo DOI: 10.5281/zenodo.6545504.

## RESULTS

### Quinolones (with the exception of moxifloxacin) induce resistance to non-quinolone antibiotics

To examine whether quinolones promote the acquisition of resistance to quinolone and non-quinolone antibiotics, we measured the frequency of antibiotic-resistant colonies after exposure to several quinolones. To do this, *E. coli* MG1655 was incubated with sub-MIC levels of quinolones (Tables S3 and S4), and resistant colonies were isolated on selection plates.

**Fig 2:**
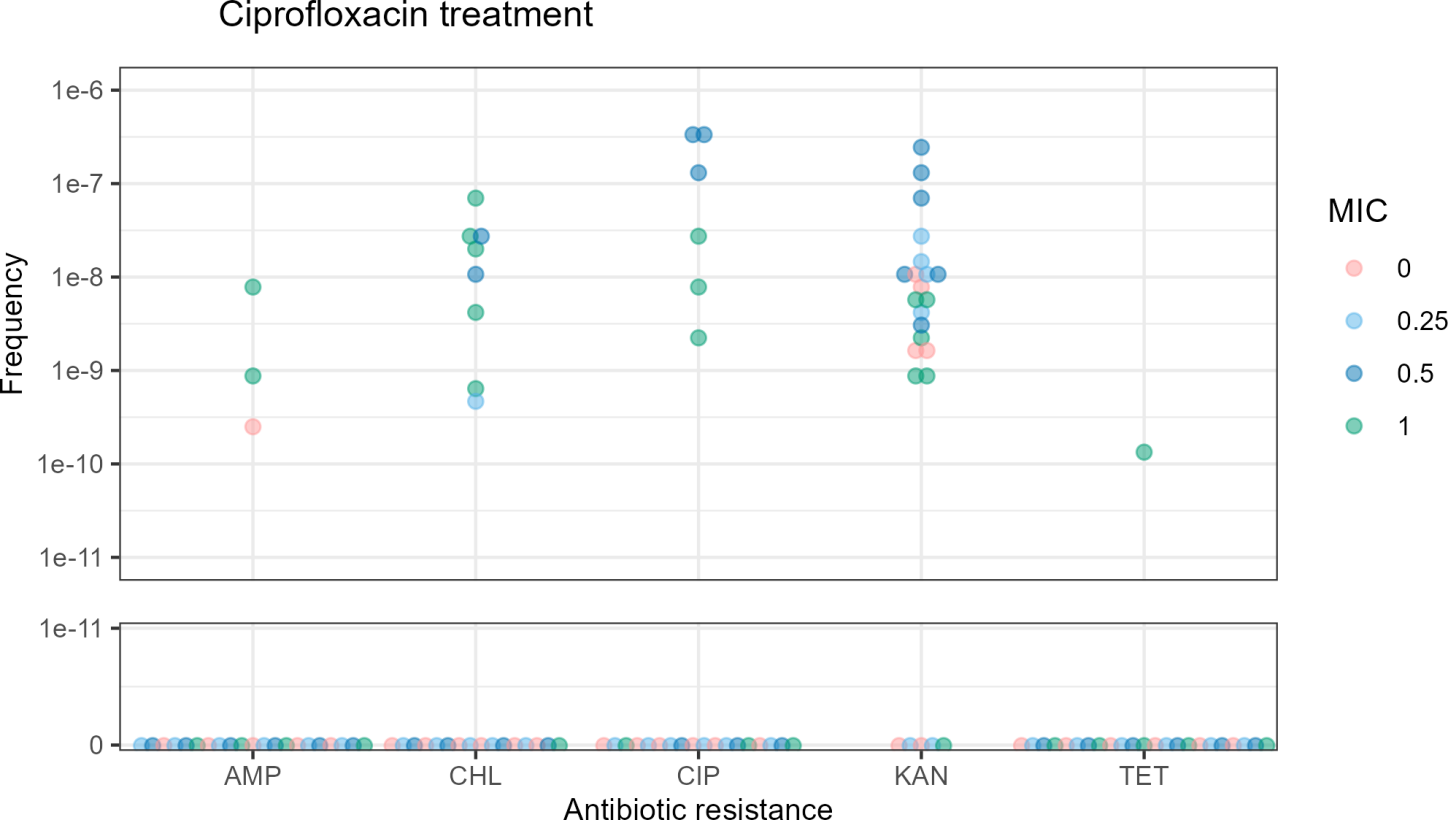
Frequency of antibiotic resistance per CFU for *E. coli* MG1655 treated with sublethal CIP over a 24 h incubation. Each dot represents a data point at 0, 0.25×, 0.5×, or 1× MIC. The X axis shows the compounds to which resistance was tested for. The Y axis represents the frequency of resistant colonies per CFU split into a linear scale from 0 to 1e-11 and a logarithmic scale from 1e-11 to 1e-6.

CIP sub-MIC treatment led to an apparent increase in the frequency of resistance to CHL, TET, AMP, and CIP itself (Fig. 2). These data were not subjected to rigorous statistical treatment; therefore, the significance is not quantified. KAN-resistant colonies were observed in untreated samples but increased in frequency following sub-MIC CIP treatment, underscoring the distinctive impact of CIP. We also tested whether other non-quinolone antibiotics such as CHL, AMP, KAN, COU, and MMC caused an increase in the frequency of antibiotic-resistant colonies (Fig. S1 and S2); sub-MIC treatment with CHL, KAN, and COU resulted in resistance to the same antibiotic administered during treatment. AMP and MMC treatment led to an increase in the frequency of resistance to them and to CHL. These results indicate that induced antibiotic resistance occurs with different types of antibiotics, but it is particularly pronounced with the quinolone CIP.

To determine the stability of CIP-induced resistance, we cultured antibiotic-resistant colonies without selection over 3 days, subculturing twice daily (data not shown). The cultures were then streaked back onto the antibiotic the original colony was identified on. Most colonies displayed stable, heritable resistance, with only a fraction (~20%) losing resistance or showing reduced resistance over time, thus minimizing the possibility of susceptibility or epigenetic factors playing a significant role.

Multidrug resistance was also assessed among CIP-treated antibiotic-resistant colonies (Table 1). We selected 27 CHL-resistant colonies and cultured them without selection and then tested their resistance to CHL as well as AMP, CIP, KAN, RIF, streptomycin, triclosan, and TMP. Among the 27 colonies, 25 retained CHL resistance, indicating its heritability. Additionally, six colonies exhibited resistance to one other antibiotic, and two were resistant to three, suggesting the presence of multidrug resistance mutations.

**TABLE 1:** Multidrug resistance test of 27 *E. coli* colonies that became resistant to CHL after CIP treatment^*a*^

Colony	Antibiotic resistance								
	AMP	CIP	CHL	KAN	RIF	STR	TET	TRI	TMP
1	No	No	No	No	No	No	No	No	No
2	No	No	No	No	No	No	No	No	No
3	No	No	Yes	No	No	No	No	Yes	Yes
4	No	No	Yes	No	No	Yes	No	No	No
5	No	No	Yes	No	No	No	Yes	No	No
6	Yes	No	Yes	No	No	No	No	No	Yes
7	No	No	Yes	No	No	No	No	Yes	No
8	No	No	Yes	No	No	No	No	Yes	Yes
9	No	No	Yes	No	No	No	Yes	No	No
10	No	No	Yes	No	No	No	No	Yes	No
11	No	No	Yes	No	No	No	No	No	No
12	No	No	Yes	No	No	No	No	No	No
13	No	No	Yes	No	No	No	No	No	No
14	No	No	Yes	No	No	No	No	No	No
15	No	No	Yes	No	No	No	No	No	No
16	No	No	Yes	No	No	No	No	No	No
17	No	No	Yes	No	No	No	No	No	No
17	No	No	Yes	No	No	No	No	No	No
19	No	No	Yes	No	No	No	No	No	No
20	No	No	Yes	No	No	No	No	No	No
21	No	No	Yes	No	No	No	No	No	No
22	No	No	Yes	No	No	No	No	No	No
23	No	No	Yes	No	No	No	No	No	No
24	No	No	Yes	No	No	No	No	No	No
25	No	No	Yes	No	No	No	No	No	No
26	No	No	Yes	No	No	No	No	No	No
27	No	No	Yes	No	No	No	No	No	No

^*a*^ Colonies were grown on LB without selection for 24 h before being grown on plates supplemented with 2× MIC of the relevant antibiotic.

**Fig 3:**
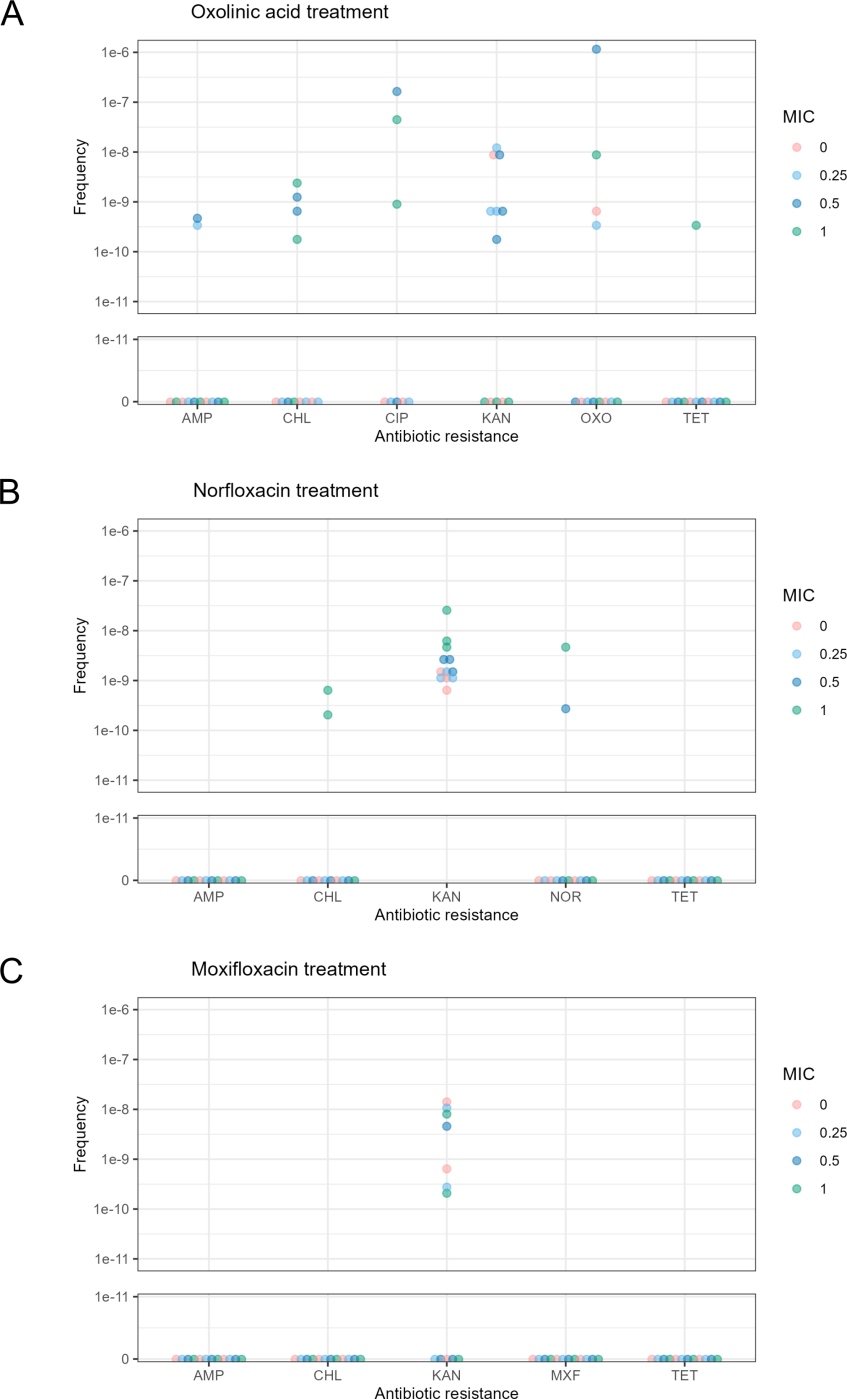
Frequency of antibiotic resistance per CFU for *E. coli* MG1655 treated with sublethal (A) OXO, (B) NOR, or (C) MXF over a 24 h incubation. Each dot represents a data point at 0, 0.25×, 0.5×, or 1× the MICs of the respective antibiotics. The X axis shows the compounds to which resistance was tested for. The Y axis represents the frequency of resistant colonies per CFU split into a linear scale from 0 to 1e-11 and a logarithmic scale from 1e-11 to 1e-6.

We also evaluated the impact of other quinolones apart from CIP: OXO, MXF, and NOR (Fig. 3). OXO treatment, like CIP, led to an increased frequency of resistance to KAN, TET, AMP, CHL, CIP, and OXO (Fig. 3A). The most common resistances observed were to KAN and CHL, with limited instances of AMP or TET resistance. NOR induced resistance to KAN, CHL, and itself (Fig. 3B). KAN resistance was present in untreated samples and became more frequent at sub-MIC levels of NOR. CHL resistance emerged at 1× MIC of NOR. In contrast, MXF resulted in increased KAN resistance within a 24-h period, but no resistance to other antibiotics, including itself, was observed (Fig. 3C). These findings show that quinolones such as OXO and NOR can also elevate the frequency of resistance to non-quinolone antibiotics; however, this is not the case for MXF, which appears to act differently.

### Moxifloxacin may act via a different mechanism

Because MXF did not induce antibiotic resistance, in contrast to other quinolones such as CIP (Fig. 3C), we wondered whether MXF might have a different target compared to CIP. *E. coli* has two enzymes that are inhibited by quinolones: DNA gyrase and DNA topo IV. To investigate whether MXF and CIP preferentially target gyrase or topo IV, we analyzed the ability of MXF and CIP to inhibit gyrase and topoisomerase IV *in vitro* and assessed the ability of these compounds to stabilize the cleavage complexes of gyrase and topoisomerase IV (Table 2). In terms of inhibition of catalytic activity, both compounds show a preference for gyrase, although MXF shows significant topo IV activity. However, in terms of the induction of cleavage, both compounds show a preference for topo IV, which was about a factor of four in the case of MXF. These results suggest the possibility that MXF could preferentially target topo IV, although it is not certain whether these relative sensitivities are reflected *in vivo*, and other options need to be borne in mind, particularly as we have only analyzed the *E. coli* enzymes and those from other bacterial species may yield different results (see Discussion).

**TABLE 2:** Comparison of the activity of CIP and MXF on the *in vitro* supercoiling activity of *E. coli* gyrase and the relaxation activity of topo IV and on the induction of cleavage for both enzymes^*a*^

	CIP (µM)	MXF (µM)	CIP/MXF
	IC_50_s		
Gyrase	0.22	0.56	0.4
Topo IV	9.6	4.3	2.2
Gyrase/topo IV	0.02	0.13	
	CC_50_s		
Gyrase	2.9	4.3	0.7
Topo IV	1.66	1.16	1.4
Gyrase/topo IV	1.74	3.7	

^*a*^ IC_50_ indicates the concentration of compound that inhibits the activity of the enzyme by 50%; CC_50_ indicates the concentration of compound that promotes 50% of the maximal DNA cleavage activity.

### Ciprofloxacin-induced antibiotic resistance decreases in a quinolone-resistant *E. coli* strain

Because DNA gyrase is the primary target of CIP (31, 58), we reasoned that CIP-induced antibiotic resistance was dependent on gyrase. To investigate the role of gyrase on CIP-induced resistance, we tested the *E. coli* strain MLS83L, which has a mutation in *gyrA* (one of the subunits of gyrase) that leads to a substantial increase in the MIC of quinolones (59).

**Fig 4:**
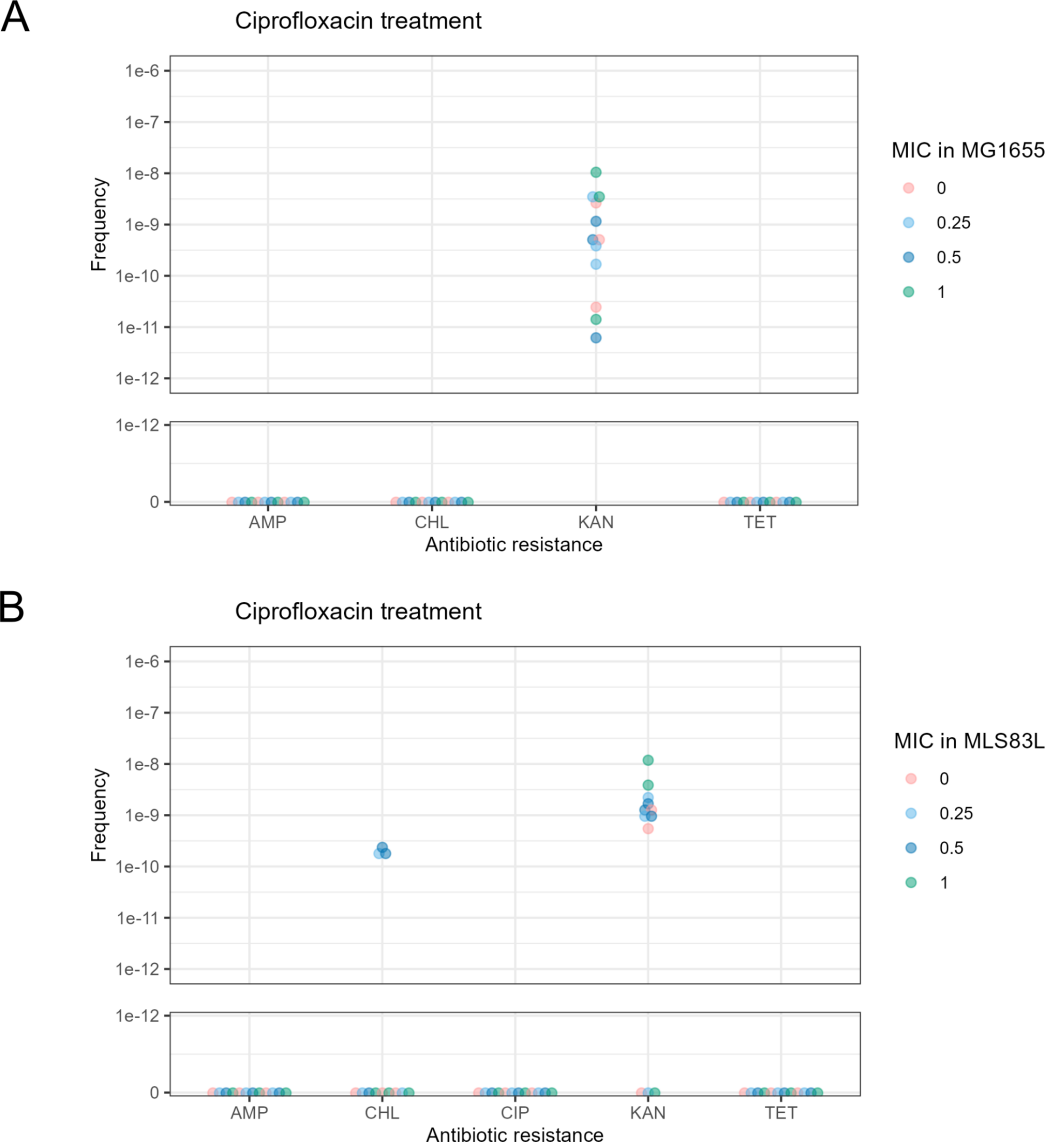
Frequency of antibiotic resistance per CFU for *E. coli* MLS83L treated with sublethal (A) CIP at 0, 0.25×, 0.5×, or 1× the MIC of MG1655 (0, 0.004, 0.008, or 0.016 µg/mL, respectively) or (B) CIP at the 0, 0.25×, 0.5×, or 1× MIC of MLS83L (0, 0.064, 128, or 0.256 µg/mL, respectively) over a 24 h incubation. Each dot represents a data point at the indicated concentration. The X axis shows the compounds to which resistance was tested. The Y axis represents the frequency of resistant colonies per CFU split into a linear scale from 0 to 1e-12 and a logarithmic scale from 1e-12 to 1e-6.

When we treated MLS83L with the same concentrations of CIP as MG1655 (i.e., 16 times less CIP than the MLS83L MIC), the observed KAN resistance was notably lower, ranging from 10 to 1,000 times lower than the resistance seen with CIP treatment, and it did not exhibit a dose-dependent increase with increasing CIP concentrations (Fig. 4A). However, when we subjected the MLS83L strain to sub-MIC levels of CIP for this strain, a recovery of KAN resistance was observed, albeit at a frequency approximately 100 times lower than the wild type (Fig. 4B). Furthermore, a dose-dependent increase in KAN resistance was noted. These results suggest that DNA gyrase plays a major role in the observed increase in resistance frequency.

### Ciprofloxacin induces resistance to chloramphenicol in a dose-dependent manner

**Fig 5:**
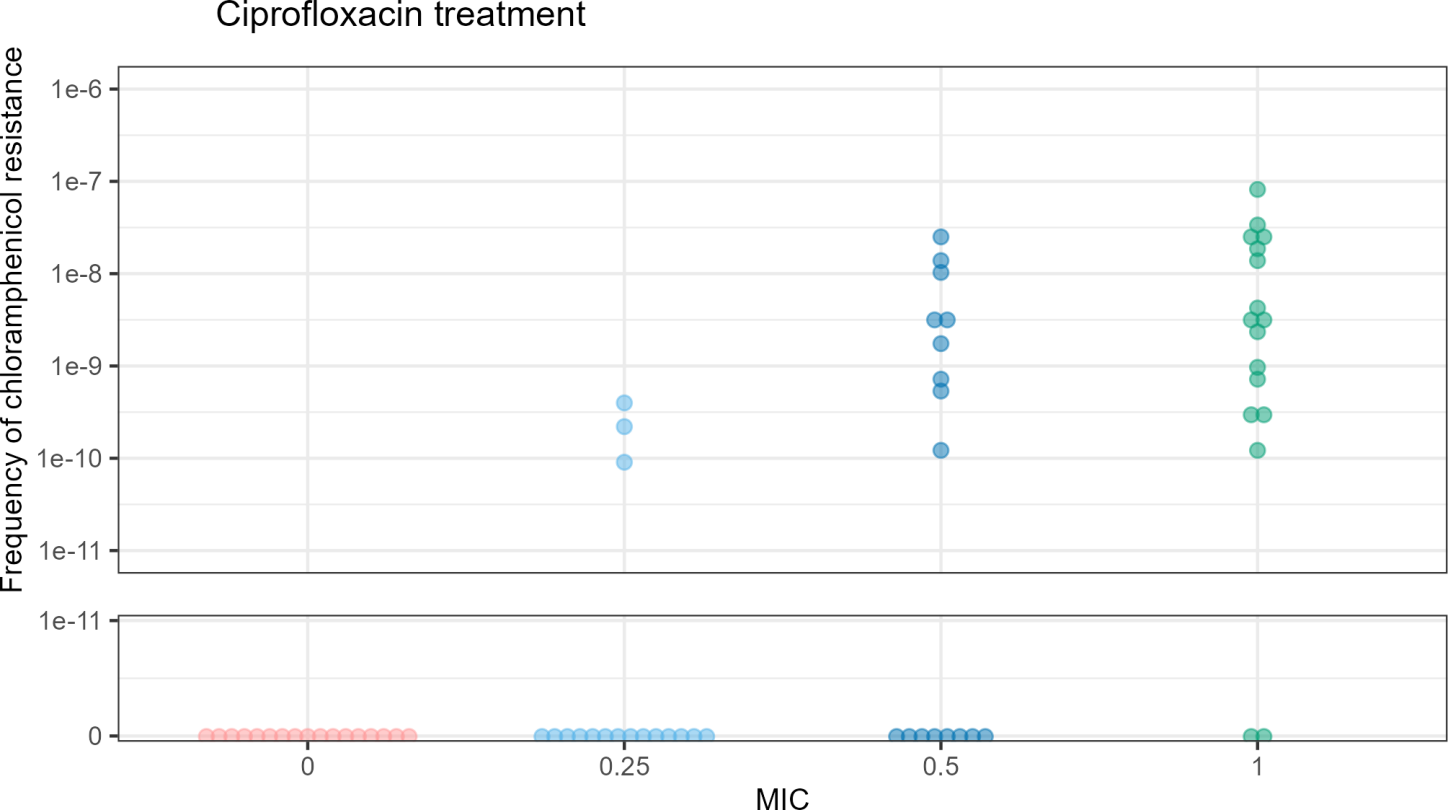
Frequency of CHL resistance after *E. coli* MG1655 was treated with 0, 0.25×, 0.5×, and 1× the MIC of CIP. The Y axis represents the frequency of CHL-resistant colonies per CFU split into a linear scale from 0 to 1e-11 and a logarithmic scale from 1e-11 to 1e-6.

To further test the CIP-induced antibiotic resistance phenomenon, we focused on the acquisition of CHL resistance after CIP treatment. Further assays (18 in total) were performed in which *E. coli* MG1655 was treated with sublethal CIP before selecting for CHL resistance. Again, no CHL-resistant colonies were seen in the untreated samples, and the frequency of CHL resistance increased in a dose-dependent manner (Fig. 5), indicating that CIP was responsible for the acquisition of CHL resistance. Interestingly, when we tested other non-quinolone antibiotics, such as AMP and MMC, we found that they also increased the frequency of CHL-resistant colonies (Fig. S1 and S2). This suggests that the induced CHL resistance may be due to a general stress response.

### Ciprofloxacin-induced chloramphenicol resistance is dependent on the SOS response but independent of SOS-activated error-prone polymerases

**Fig 6:**
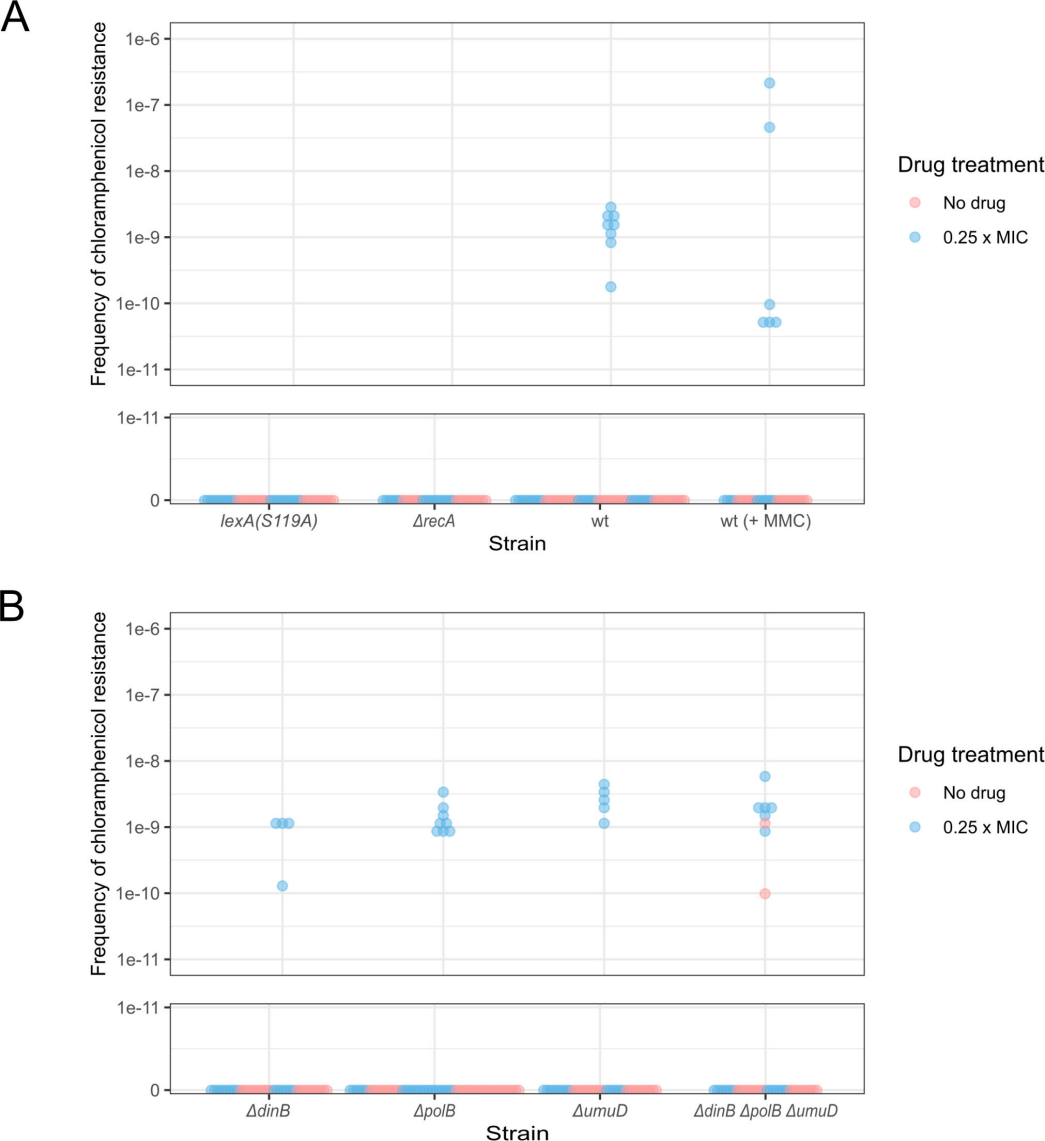
Frequency of CHL-resistant bacteria obtained after the incubation with or without a drug of (A) WT *E. coli* and mutants that cannot activate the SOS response and (B) *E. coli* strains lacking the SOS response error-prone polymerases. Each dot represents an independent colony that was grown with (blue dot) or without a drug (pink dot). The drug used was CIP unless it is stated in the X axis. The amount of drug used was 0.25× MIC for the respective strain tested. The Y axis represents the frequency of resistant colonies per CFU split into a linear scale from 0 to 1e-11 and a logarithmic scale from 1e-11 to 1e-6.

As MMC, CIP, OXO, and NOR induced resistance to CHL (Fig. 2; Fig. S2) and are activators of the SOS response (21, 60), we hypothesized that the SOS response was involved in the CIP-induced CHL resistance. To test this, we measured the frequency of CHL-resistant colonies after exposure to low levels of CIP or MMC (Fig. 6). When wild-type *E. coli* MG1655 was exposed to CIP or MMC, CHL-resistant colonies were obtained. However, when cells were exposed to CIP without the SOS response activator RecA, or with a mutation in the SOS response repressor LexA, there were no CHL-resistant colonies visible (Fig. 6A). These results indicate that the activation of the SOS response is crucial in the acquisition of CHL resistance after CIP exposure.

We also investigated whether the error-prone polymerases (*dinB*, *polB*, and *umuDC*) were responsible for the acquisition of CIP-induced CHL resistance (Fig. 6B). The error-prone polymerases are upregulated during SOS response and can cause mutations (61, 62). When the three error-prone polymerases were deleted, both individually and together, there was no clear difference in the frequency of CHL-resistant colonies. This suggests that these polymerases are not necessary for the acquisition of CIP-induced CHL resistance.

### Whole-genome sequencing of ciprofloxacin-induced antibiotic-resistant strains shows a wide variety of mutations

To investigate whether CIP-induced antibiotic resistance was due to mutations, we analyzed the whole-genome sequences of CIP-induced antibiotic-resistant isolates; we analyzed a total of 35, including 19 resistant and 16 non-selected strains (Table 3; Table S5). Non-selected isolates were derived from LB-only plates that had been exposed to 0.25×, 0.5×, or 1× MIC CIP, but without secondary selection. As controls, two parental strains from LB-only plates were included.

**TABLE 3:** Unique variants and mutations identified in whole-genome sequencing of 35 *E. coli* MG1655 strains from the QIAR assay and the MFA^*a*^

Secondary selection	Sample	Strain	mdfA	marR	e14	icd	gyrA	fusA	clpX	lon	Non-coding^*b*^	fetB	nuoF	suhB	ung	dppD	yibA	cyaA	cpxA	frdD	leuP
No incubation	Reference	WT														SNP					
	Reference	*ΔdinBΔpolBΔumuD*																			
No selection	0 CIP	WT																			
	0 CIP	WT																			
	0 CIP	WT																			
	0 CIP	WT																			
	0.25× CIP	WT																			
	0.25× CIP	WT																			
	0.25× CIP	WT													SNP						
	0.25× MMC	WT																			
	0.25× CIP	*ΔdinB*	Amplification														SNP				
	0.25× CIP	*ΔdinBΔpolBΔumuD*																			
	0.5× CIP	WT																			
	0.5× CIP	WT																			
	1× CIP	WT					SNP														
	1× CIP	WT					SNP														
AMP selection	0.5× CIP	WT																		SNP	
TeT selection	1× CIP	WT		SNP					DEL	DEL	INS										
CIP selection	1× CIP	WT					SNP							INS							
	1× CIP	WT		SNP			SNP														
CHL selection	0.25× CIP	WT		INS																	
	0.25× CIP	WT																			
	0.5× CIP	WT			DEL	SNP															
	0.5× CIP	WT	Amplification		DEL	SNP															
	0.5× CIP	WT	Amplification																		
	1× CIP	WT		DEL																	
	1× CIP	WT	Amplification	SNP	DEL																
	1× CIP	WT	SNP^*c*^	SNP																	
	0.25× MMC	WT	Amplification		DEL	SNP															
	0.25× MMC	WT		DEL	DEL	SNP															
	0.25× CIP	*ΔdinB*	Amplification	DEL																	
	0.25× CIP	*ΔdinBΔpolBΔumuD*	Amplification																		
KAN selection	0 CIP	WT						SNP				SNP							SNP		
	0.5× CIP	WT						SNP					SNP								DEL
	1× CIP	WT		SNP				INS										DEL			

^*a*^ 0.25×, 0.5×, and 1× MIC CIP is 0.004, 0.008, and 0.016 µg/mL respectively.

^*b*^ bp 468358.

^*c*^ Single nucleotide polymorphism (SNP) mutation in noncoding region between promoter and gene.

Analysis of the mutations revealed that non-selected isolates exhibited few mutations, primarily single nucleotide polymorphisms (SNPs), with an exception of the error-prone polymerase mutant *ΔdinB* strain that also had an amplification. In contrast, resistant isolates (those that had been subjected to secondary selection) showed a diverse array of mutations, including SNPs, amplifications, and deletions. Most colonies exposed to 1× MIC CIP had mutations in *gyrA*, resulting in an amino acid substitution at position S83 (bp 2339173), that confers CIP resistance (63). One CIP-resistant isolate also had an SNP in *marR*, a transcriptional repressor of the *marRAB* operon, which negatively regulates the transcription of efflux pumps involved in antibiotic resistance (64, 65).

AMP resistance was linked to an A > T transversion (bp 4379035) in the *ampC* promoter region. This mutation might lead to increased *ampC* gene expression, which encodes a β-lactamase that leads to AMP resistance (66–68). TET resistance appeared to be the outcome of two mutations, a deletion in the *lon* gene (∆A at bp 459223) and a C > T transition mutation in *marR* (bp 1619468). The *lon* gene encodes a protease involved in the evolution to low levels of multidrug resistance, as is the marR transcriptional regulator (64, 69, 70). KAN resistance correlated with mutations in the elongation factor G *fusA*, including insertions and SNPs. Mutations in *fusA* resulting in KAN resistance have been previously reported in *E. coli* (71, 72). Additionally, we found an A > T transversion mutation (bp 4104443) in the kinase *cpxA* and a single bp deletion in the adenylate cyclase *cyaA*. Mutations in *cpxA* and *cyaA* have been associated with multidrug resistance (71, 73). Moreover, a 148 bp deletion in *leuP* (one of eight leucine tRNAs) was observed in one isolate, although no prior associations of this gene with antibiotic resistance have been reported.

CHL-resistant isolates include *marR* and *icd* mutations, *mdfA* amplifications, and the deletion of the e14 prophage. *mfdA* encodes a transmembrane ATPase multidrug efflux pump (74) that was initially identified by its ability to confer resistance to CHL when overexpressed (75). e14 is a cryptic prophage in *E. coli* MG1655 known to excise from the genome when the SOS response is induced (76, 77). The *icd* mutation, which encodes for isocitrate dehydrogenase (enzyme that participates in the tricarboxylic acid cycle), seen in the CHL-resistant isolates, is mostly synonymous with the e14 deletion. Mutations in *icd* have been associated with antibiotic resistance (78), and, in MG1655, the e14 prophage overlaps with the *icd* gene. The excision of e14 is known to affect the coding sequence of the gene without the activity of *icd* being affected (79). Therefore, mutations in *marR*, *mdfA*, and e14, but not in *icd*, might be responsible for CHL resistance. Interestingly, both CIP- and MMC-treated isolates had mutations in the same loci (*marR*, e14, and *mdfA*), suggesting potential cross-resistance mechanisms.

Since we identified mutations in *marR* in 9 out of the 19 sequenced resistant isolates (Table 3), we replicated one of these mutations, a partial deletion of *marR*, in a WT *E. coli* strain (Fig. S3). Our results demonstrated that the *marR* mutation led to an eightfold increase in the MIC of CHL compared to the WT strain. These results support the *marR* mutation as playing a significant role in conferring CHL resistance.

In summary, the analysis of the whole-genome sequences of CIP-induced antibiotic-resistant isolates suggests that various mutations (especially in *marR*) are responsible for the acquired antibiotic resistance.

## DISCUSSION

The advent of AMR has severely threatened the usefulness of antibiotics and presented us with the prospect of a return to the pre-antibiotic era. Over and above AMR, there is evidence that treatment with low doses (sub-MIC) of certain antibiotics, for example, the fluoroquinolones, leads to the induction of resistance to other, unrelated antibiotics. Here, we have investigated this phenomenon and found that QIAR occurs for several quinolones (CIP, NOR, and OXO) but surprisingly not for MXF. The nature of the resistance mutations is diverse and includes point mutations, insertions, and deletions. QIAR requires the SOS response but is not a consequence of repair by error-prone polymerases, such as DinB and PolB. Our data support the involvement of DNA gyrase (the principal quinolone target in *E. coli*) in mediating the QIAR process.

### Ciprofloxacin induces antibiotic resistance to non-quinolone antibiotics in wild-type *E. coli* (MG1655)

When *E. coli* MG1655 was treated with sublethal concentrations of CIP, there was an increase in resistance to non-quinolone antibiotics. The increase in resistance appears to be dose-dependent, but the frequency with which this resistance appears differs depending on the antibiotic selected. Resistance to several non-quinolones was observed, including KAN, CHL, TET, and AMP. The most common resistance seen was to KAN and CHL with fewer occurrences to AMP and TET. Increased frequencies of resistance with sublethal CIP treatment have been demonstrated previously and concur with the results seen here. Gullberg et al. (11) (reviewed in reference [12]) demonstrated an increase in fluoroquinolone resistance when *E. coli* was incubated with 0.1× MIC of CIP.

Resistance to KAN, CHL, AMP, and TET generally appeared to be maintained over generations, and when these isolates were sent for WGS, genetic explanations for the resistance were evident. The AMP resistance was identified as an SNP mutation in the *ampC* promoter region, the TET resistance was attributed to two mutations in *lon* and *marR*, the KAN resistance was due to a mutation in the *fusA* gene, and the CHL resistance was either attributed to mutations in *marR* or amplifications over the *mfdA* gene.

The need for two mutations to induce TET resistance may explain why only low levels of TET resistance have been seen in these assays as the probability of the formation of two mutations is much lower than that of just one. Mutations in *lon* have also been demonstrated in *Pseudomonas aeruginosa* when treated with sublethal CIP (80). Lon protease has been suggested to be involved in the repair of quinolone-induced DNA damage (81).

One of the most common mutations identified that was associated with the CHL and TET resistance, which was also identified in a KAN- and a CIP-resistant isolate, was in *marR*. The Mar operon (multiple antibiotic resistance) contains three genes *marR*, *marA*, and *marB*. MarR is the transcriptional repressor of *marA* and *marB,* and derepression of these results in multidrug resistance through MarA, which regulates a number of efflux pumps and porins. Mutations in *marR* are known to give rise to resistance to various antibiotics and antibacterials, including TET, CHL, and FQs (64, 82, 83). Considering these mutations should give multidrug resistance, resistance to more than just CHL should have been seen; however, there may have been a decreased susceptibility of these strains to the other antibiotics that was not visible at the concentrations of the antibiotics used. Indeed, the TET-resistant isolate had a *marR* mutation that is thought to have contributed to the resistance. The high number of *marR* mutations obtained, particularly with treatment at 1× MIC CIP, may imply that the resistance is induced to reduce the effects of the CIP, and the CHL resistance may be a secondary effect. Specific resistance to fluoroquinolones mediated by *marOR* mutations has been identified in *E. coli* isolated from patients with urinary tract infections that were treated with various fluoroquinolones (84) and demonstrated under treatment with sub-MIC NOR (85).

Alongside the *marR* mutations, *mfdA*, which encodes a transmembrane ATPase multi-drug efflux pump (74), was also associated with CHL resistance. This gene has also been found to increase resistance to a number of molecules as well as several clinically relevant antibiotics including fluoroquinolones (74); however, clinically significant increases in resistance to fluoroquinolones have been shown to require over-expression of more than one efflux pump, such as *mdfA* and *acrAB* (86). With one of the CHL-resistant isolates from the 0.5× MIC CIP treatment and the CHL-resistant isolate from the *ΔdinBΔpolBΔumuD* strain, this amplification of the *mdfA* gene is the only genetic explanation for the CHL resistance observed. The amplification appears to have increased the read coverage of this gene, ninefold in the 1× MIC CHL-resistant isolate, fivefold in the 0.5× MIC CHL-resistant isolate, and 10-fold in the *ΔdinB* CHL-resistant isolate.

Gene duplications and amplifications (GDAs) have been suggested to be found frequently in bacterial populations (including those not under any selection [87, 88]) and have previously been implicated in resistance in a number of bacterial species (89–93) including some clinical isolates (94, 95). In particular, MG1655 was shown to have gene duplications of 12–140 kb after short-term laboratory evolution experiments in lactate minimal media where a one- to fourfold increase in coverage in their assemblies in areas of the amplifications was found (90). Slager et al. (92) showed that antibiotics that target DNA replication increased gene dosage near *oriC* in *Streptococcus pneunomiae* that was a result of stalled replication forks and refiring of the replication origin. Tandem duplications of 98 kb were found in MRSA clinical isolates as well as 20 kb amplifications that included *mprF*, which confers resistance to antimicrobial peptides, were identified in vancomycin-intermediate *S. aureus* (94). Due to GDA being found in bacteria under no particular selection, it has been suggested that these mutations are often the first selected for when a selection pressure is applied (such as antibiotics) (88, 89). Further to this, GDA may be a short-term solution (as GDAs have been shown to generally be unstable in a population [91]) to selection pressure until a stable mutation is established. Thus, GDAs may facilitate antibiotic resistance by allowing populations time to accumulate point mutations that may confer stable resistance (89). GDAs have been demonstrated to arise through RecA-dependent and -independent routes (89). The RecA-dependent routes include non-equal homologous recombination between long direct repeats (96), transposable or insertion elements (97), or repeat regions (98). The RecA-independent routes include DNA secondary structure (99) or crossovers between sister chromatids (100) that may direct recombination between direct repeats or through illegitimate recombination by gyrase (101). Alternatively, they could happen as a result of double-strand DNA breaks (DSBs) and rolling circle replication (89). Here, it appears as though the GDA across the *mfdA* gene may have been present in the population. This was then selected for by the treatment with sublethal CIP, and under stronger selection pressure, either the GDA increased or other mutations accumulated to overcome the selection pressure.

### Antibiotic resistance is observed in wild-type MG1655 treated with other quinolones but is less common with non-quinolone antibiotics

When the QIAR assay was performed with KAN or AMP as the treatment antibiotic, KAN-resistant colonies were isolated when treated with KAN and isolates resistant to AMP, CHL, and CIP after treatment with AMP. However, the frequencies of resistance seen with AMP were about 100-fold lower than that seen with CIP. Other studies have shown a rise in multidrug resistance after incubation with sublethal levels of AMP and increased mutation rates when treated with AMP or KAN (42). β-lactam antibiotics, such as AMP, are thought to increase mutations either via induction of the RpoS regulon, leading to an increase in resistance to fosfomycin, TET, and RIF (19), or via induction of the SOS response (102, 103); both of these pathways can result in the upregulation of the error-prone polymerase Pol IV (*dinB*) (104). The discrepancies between this work and previous work may be down to experimental differences, such as with Kohanski et al. (42), who were looking for an increase in MIC (as opposed to resistant colonies at a fixed selection concentration), and the longer incubation times (37°C for 48 h vs 22 h at 37°C with a further incubation at 22°C for 18 h). This is also true for study conducted by Gutierrez et al. (19) who also only scored colonies after a 48 h incubation at 37°C.

To ascertain whether the increase in resistance was due to quinolones specifically or whether it was down to the inhibition of gyrase, a non-quinolone gyrase inhibitor was tested. Coumermycin A1 is an aminocoumarin antibiotic that targets GyrB and competitively inhibits the ATPase reaction (105, 106). Resistance to COU and KAN was observed, but the KAN resistance was seen in untreated samples and at very low levels of COU, and the frequency of KAN resistance was 10-fold lower than that seen with CIP. COU has also been shown to reduce the frequency of illegitimate recombination in *E. coli* (107). This all implies that merely inhibiting DNA gyrase does not cause an increase in resistance indicating that QIAR is likely to be due to the stabilization of the gyrase-DNA cleavage complex.

The idea that the stabilization of cleaved complexes may be important in the induction of resistance raised the question on whether DSBs alone can be responsible for the increase in resistance. MMC is a DNA minor groove intercalator that crosslinks DNA strands and induces DSBs (108, 109). When *E. coli* MG1655 was treated with MMC, resistance was observed to CHL and CIP. This implies that DSBs are linked to the generation of resistance. This is unsurprising as in *E. coli*, the accumulation of DSBs is linked to the induction of the SOS response (110), and MMC has been shown to be a strong inducer of the SOS response (60, 111). However, the breadth of resistance seen with treatment with sublethal CIP was not observed with MMC, which may indicate that another process is involved.

Other quinolones across the quinolone “generations” were also tested for their ability to increase resistance to non-quinolone antibiotics. OXO is a first-generation quinolone (112–114) that lacks the C6-fluorine. CIP and NOR are second-generation fluoroquinolones, while MXF is a fourth-generation compound (31, 114, 115). The lethality and pathway of killing by quinolones have been demonstrated to be dependent on which generation or type of quinolone used (31, 116). OXO showed very similar results to those obtained with CIP with resistance observed against all antibiotics tested. This discontinued antibiotic has previously been found to increase illegitimate recombination mediated by DNA gyrase (107), which may be a mechanism of introducing mutations.

NOR has been used extensively to study the effect that subinhibitory concentrations of antibiotics and antibacterials have on resistance (42, 85, 117, 118), and this is despite NOR having been demonstrated to affect killing in cells by a different mechanism than that of most other fluoroquinolones (81, 119). Specifically, killing with NOR appears to rely on continued protein synthesis, and it has been shown to upregulate ROS (120). Resistance to CHL and KAN was seen with NOR, although not to the same extent as with OXO and CIP.

A potentially interesting finding was that little increase in resistance was observed with MXF. Although KAN resistance was seen, the highest frequency of resistance observed was with an untreated sample. It has been reported that MXF inhibits *E. coli* gyrase with a lower IC_50_ than CIP (i.e., it takes less MXF to inhibit the enzyme by 50% than CIP); however, MXF was also shown in the same study to have a mutation rate 100-fold less than that seen with CIP at 4× MIC (121). MXF has been shown to target both topo IV and DNA gyrase equally in *Streptococcus pneumoniae* (122). Additionally, in *S. aureus*, gatifloxacin, which like MXF is an 8-methoxyfluoroquinolone, was also shown to target both topo IV and DNA gyrase equally (123). MXF has also been shown to increase the selection of resistant mutants in comparison to the β-lactam ceftriaxone when tested against *Streptococcus pneumoniae*; although in this same study, it had the lowest selection frequencies in comparison to the other quinolones tested (124). It appears that there are some potential anomalies with MXF. In our work, we found that it was the only quinolone tested that did not induce significant resistance to other antibiotics. Our *in vitro* assays showed that the potencies of MXF in cleavage assays for *E. coli* gyrase and topo IV suggested a preference for topo IV by a factor of ~4 (Table 2). Therefore, it is possible that the cause of the reduced QIAR effect with MXF is its preferential targeting to topo IV, suggesting that arresting the topo IV-cleavage complex may not have the same consequence, in terms of mutagenesis, that arrest of the gyrase cleavage complex does. Another possibility is that its ability to target both enzymes (gyrase and topo IV) in *E. coli* may more readily lead to cell death avoiding the recombinogenic events that lead to QIAR. This phenomenon warrants future investigation. To consolidate these findings, work with other bacterial species would be required.

The differences in the frequencies of resistance seen with the different quinolones may be linked to the differences in their killing pathways. Based on the fact that here the bacteria were treated with sublethal concentrations of quinolones, it is likely that cell death follows a slower pathway than the rapid cell death that has been linked with the accumulation of ROS (120). This slow pathway of killing is thought to be as a result of the release of DSBs from DNA gyrase (31, 119, 125), but this slower path also allows for DSB repair and other repair mechanisms that may be upregulated within the SOS response.

### Frequency of antibiotic resistance is lower in MG1655 strain with quinolone-resistant DNA gyrase

In light of the increase in resistance seen with OXO, CIP, and NOR, a quinolone-resistant MG1655 isogenic strain MLS83L was tested (59). Here, when QIAR was tested at the CIP MIC of MG1655, there was a significant reduction in the resistance seen. When the MIC of MLS83L, which is 16× higher than the wild-type MIC, was used, there was an increase in the frequency of KAN resistance per CFU and a return of the CHL resistance, although the frequencies of resistance were lower than those recorded for MG1655. This implies that DNA gyrase, rather than topo IV, is responsible for the increase in the resistance. High-level resistance on the other hand has been shown to be dependent on multiple mutations, including efflux mutations and mutations in topo IV (84, 126).

### Quinolone-induced chloramphenicol resistance is dependent on SOS response but not error-prone polymerases

The SOS response is a bacterial response to DNA damage, and it is regulated by RecA and LexA. During normal growth, the SOS genes are negatively regulated by the repressor LexA. Activation of the SOS genes occurs after DNA damage and an increase in the presence of single-stranded DNA, to which RecA, binds followed by interaction of RecA with LexA to facilitate the self-cleavage of LexA (127). The autocatalytic proteolysis of LexA results in the derepression of the SOS regulon that controls the expression of genes including the ones that encode error-prone polymerases which can cause mutations (61, 62). Studies that have looked at the effect of sublethal antibiotics on the development of mutations have shown that genes involved in the SOS response are responsible for the elevated mutation rate under sublethal treatment with CIP, including *recA* which, when deleted, showed reduced mutation rates when incubated with 0.5× MIC of CIP (18). The cleavage of LexA and the concomitant derepression of the three error-prone polymerases *polB*, *dinB*, and *umuCD* have also been shown to be necessary for the increase in mutation caused by sublethal treatment with CIP in *E. coli*, *P. aeruginosa*, and *S. aureus* (43, 44, 53, 128). In this study, no CHL-resistant isolates were identified in the *ΔrecA* and *lexA*(S199A) strains using the MFA (Fig. 6). These strains are not able to activate the SOS response, which indicates that activation of the SOS response is important in the induction of CHL resistance. This finding concurs with the literature cited above and emphasizes the suggestion that targeting RecA and LexA in combination antimicrobial therapy could reduce resistance (128–133).

Where our data differs from other work is that we found error-prone polymerases were not required for CHL resistance. The SOS-inducible error-prone polymerases cause point mutations and have been associated with the increase in mutation rate and frequencies observed when bacteria are treated with quinolones (43, 44, 53, 128). Here, when the error-prone polymerase knockout strains (*ΔdinB* and *ΔdinBΔpolBΔumuD*) were treated with CIP, CHL-resistant isolates were still found. However, when two of these isolates were sent for WGS, no SNP mutations were seen. Furthermore, in the *ΔdinBΔpolBΔumuD* CHL-resistant isolate, there were no deletion mutations either. Similar results were obtained by Song et al. (54) in a more exhaustive study looking at the mutations caused by NOR. They found that NOR treatment increased point mutations and indels in the *rpoB* or *thyA* gene; however, in cells lacking *dinB*, they only found deletions. Pribis et al. (13) found a reduction in the mutation rate in error-prone polymerase-deficient strains treated with CIP. In the *ΔdinBΔpolBΔumuD* strain, the only explanation for the CHL resistance was an increase in the gene dosage of the *mdfA* gene caused by an amplification over the area. The lack of SNPs and small indels suggests that these types of mutation are induced by the SOS-inducible error-prone polymerases, but it also illustrates the multiple pathways to resistance. Pribis et al. (13) have demonstrated that the increased mutation rates seen after treatment with CIP are a result of RpoS induction by ROS. Separately, others have shown that sublethal treatment with CIP increases recombination between homologous sequences (14) and between non-homologous sequences independently of the SOS response (15), implying that treatment with CIP is enough to cause significant chromosomal modifications.

Although the SNPs and small indels observed can be attributed to the error-prone polymerases, they do not explain the larger deletions seen here. Sublethal treatment with fluoroquinolones has been demonstrated to induce small deletions (<19 bp) in *E. coli* (54, 85), and these were suggested to be independent of error-prone polymerases. Such polymerases have been shown to introduce spontaneous deletions in *Salmonella enterica* Typhimurium as a result of DSB repair. However, the size of these deletions was not discussed (134).

The excision of the e14 prophage can be attributed to the induction of the SOS response, and previous excision of the prophage during treatment with sublethal NOR has been demonstrated (85). The e14 prophage is a defective prophage with important phage-related functional genes having been deleted since integration (135). The e14 prophage has been shown to excise from the genome if the SOS response is induced (76, 136). The other genomic deletions observed in this study are more difficult to explain, and to the best of our knowledge, no large deletions (>100 bp) have been reported as a response to sublethal treatment with CIP previously.

From this work, it is apparent that the induction of resistance caused by sublethal treatment by quinolones is multi-faceted. It is dependent on the SOS response but does not require the error-prone polymerases (Fig. 6). However, when present, these polymerases contribute to the single-point mutations leading to CHL resistance, but resistance to CHL caused by the increased gene dosage of *mdfA* suggests that resistance can also arise through other, error-prone polymerase-independent pathways. However, the amplifications associated with this form of CHL resistance were also seen in a non-resistant isolate. This suggests that treatment with sublethal CIP may merely be selecting for resistant bacteria, or it may potentiate bacteria to become resistant when exposed to a secondary antibiotic (secondary selection pressure). To the best of our knowledge, there is no evidence in the literature that this has been directly tested; however, Torres-Barceló et al. (137) have argued that the induction of the SOS-induced response by CIP does not increase evolvability, but competitive fitness, implying that for mutations to become fixed in the population, continued selection is needed (e.g., a secondary selection pressure). This was also demonstrated by Gullberg et al. (11) who showed in competition experiments that continued selection with sublethal antibiotics gave individuals with resistance mutations a competitive advantage over those without. This study ultimately demonstrates that when populations of bacteria are exposed to sublethal CIP, mutations are induced via the SOS response which are selected for during a secondary selection. However, treatment with sublethal CIP also selects for natural resistances within the population. This natural resistance also grants individuals in the population time to accumulate mutations so that when a secondary, stronger selection is imposed, those strains have a competitive advantage.

### Overall significance of this work

We have undertaken a systematic study of the phenomenon of quinolone-induced antibiotic resistance and shown that treatment of *E. coli* with sublethal concentrations of most quinolones can lead to resistance to other, unrelated, antibiotics, a feature that must be borne in mind when treating humans and animals with these drugs, particularly in unregulated settings. We have shown that several different types of mutation can result, and these can correlate with the antibiotic resistance found. DNA gyrase is strongly implicated in the mechanism of this resistance, which is likely linked to its ability to form transient double-stranded breaks in DNA. More detailed mechanistic work will be required to fully understand this phenomenon.

## SUPPLEMENTAL MATERIAL

Supplemental material aac.00997-24-s0001.docx Supplemental methods and results, Tables S1 to S5, and Fig. S1 to S3.

ASM does not own the copyrights to Supplemental Material that may be linked to, or accessed through, an article. The authors have granted ASM a non-exclusive, world-wide license to publish the Supplemental Material files. Please contact the corresponding author directly for reuse.
