# Supplementary material for: Insights into antibiotic resistance promoted by quinolone exposure
Source: Antimicrob Agents Chemother. 2024 Nov 26;69(1):e00997-24. doi: 10.1128/aac.00997-24 (PMC11784200; doi:10.1128/aac.00997-24)
Supplement: Supplemental material — Supplemental methods and results, Tables S1 to S5, and Fig. S1 to S3. [file aac.00997-24-s0001.docx]

**Supplementary Materials**

**Insights into antibiotic resistance promoted by quinolone exposure**

Natassja G BUSH^1,2,†^, Isabel DIEZ-SANTOS^1,3,†^, Pilla SANKARA KRISHNA^1,2^, Bernardo CLAVIJO^4,5^, and Anthony Maxwell^1,6,*^

^1^Department of Biological Chemistry, John Innes Centre, Norwich Research Park, Norwich, NR4 7UH, UK

^2^Present Address: Inspiralis Ltd., Innovation Centre, Norwich Research Park, Colney Lane, Norwich, NR4 7GJ, UK

^3^Present Address: Earlham Institute, Norwich Research Park, Norwich, NR4 7UZ, UK

^4^Earlham Institute, Norwich Research Park, Norwich, NR4 7UZ, UK

^5^Present Address: Illumina Centre, 19 Granta Park, Cambridge CB21 6DF, UK

^6^Department of Molecular Microbiology, John Innes Centre, Norwich Research Park, Norwich, NR4 7UH, UK

*Corresponding author. Tel: +44 1603 450771; email: [tony.maxwell@jic.ac.uk](mailto:tony.maxwell@jic.ac.uk)

^†^These authors contributed equally to the work

**Supplementary Methods**

Table S1. *E. coli* strains, bacteriophage and plasmids.

| ***E. coli* strain** | **Relevant genotype** | **Source** |
| --- | --- | --- |
| MG1655 | K-12 F- λ- *ilvG*- *rfb*-50 *rph*-1 | *E. coli* Genetic Stock Center |
| *ΔrecA* | MG1655, *recA*::Kan^R^ | Dr. Susan Rosenberg |
| *lexA(S119A)* | MG1655, *lexA(S119A)* | This project |
| *ΔdinB* | MG1655, *ΔdinB* | This project |
| *ΔpolB* | MG1655, *ΔpolB* | This project |
| *ΔdinB ΔpolB* | MG1655, *ΔdinB ΔpolB* | This project |
| *ΔdinB ΔumuD* | MG1655, *ΔdinB ΔumuD* | This project |
| *ΔpolB ΔumuD* | MG1655, *ΔpolB ΔumuD* | This project |
| *ΔdinB ΔpolB ΔumuD* | MG1655, *ΔdinB ΔpolB ΔumuD* | This project |
| *marR*::Kan^R^ | MG1655, *marR*::Kan^R^ | This project |
| *ΔmarR* | MG1655, *ΔmarR* | This project |
| *marR170*::Kan^R^ | MG1655, *marR170*::Kan^R^ | This project |
| *marR170* | MG1655, *marR170* | This project |
| EcNR2 | MG1655, *bio-,* λ-Red genes, *mutS–*, Amp^R^ , Cam^R^ | Addgene |
| **Bacteriophage** | **Relevant genotype** | **Source** |
| P1 | *E. coli* bacteriophage P1 (ATCCr 25404-B1^tm^) | Dr. Jessica Blair |
| **Plasmid** | **Relevant features** |  |
| pKD4 | Kan^R^, FRT sites | *E. coli* Genetic Stock Center |
| pKD3 | Cam^R^, FRT sites | *E. coli* Genetic Stock Center |
| pKD46 | Amp^R^, λ-Red genes (*exo*, *beta*, *gam*) with paraB inducible promoter, *repA101ts* | *E. coli* Genetic Stock Center |
| pCP20 | *FLP*, Amp^R^, Cam^R^, *repA101ts* | *E. coli* Genetic Stock Center |
| pUC19 | Amp^R^ | Sigma |

Kan^R^- kanamycin resistance, Amp^R^- ampicillin resistance, Cam^R^- chloramphenicol resistance, FRT- flippase recognition target, *FLP* - flippase.

Table S2. List of primers.

| **Name** | **5'-3' sequence** | **Comments** |
| --- | --- | --- |
| recA-H1_FW | ATTGACTATCCGGTATTACCCGGCA | Confirmation of *recA* deletion |
| recA-H2_RV | GCCGCAGATGCGACCCTTGTGTATC | Confirmation of *recA* deletion |
| dinB-pKD4_FW | TCTCAAACCCTGAAATCACTGTATACTTTACCAGTGTTGAGAGGTGAGCAGTGTAGGCTGGAGCTGCTTC | Amplification fragment containing FRT and Kan^R^ |
| dinB-pKD4_RV | CAGTGATACCCTCATAATAATGCACACCAGAATATACATAATAGTATACAATGGGAATTAGCCATGGTCC | Amplification fragment containing FRT and Kan^R^ |
| polB-pKD4_FW | CAAACGAAACCAGGCTATACTCAAGCCTGGTTTTTTGATGGATTTTCAGCGTGTAGGCTGGAGCTGCTTC | Amplification fragment containing FRT and Kan^R^ |
| polB-pKD4_RV | AAAGCATTCGTCACGCATCAAAATGGTATCTGGCGAACTCTTTTTTTTGCATGGGAATTAGCCATGGTCC | Amplification fragment containing FRT and Kan^R^ |
| umuD-pkD4_FW | CAAGAACAGACTACTGTATATAAAAACAGTATAACTTCAGGCAGATTATTGTGTAGGCTGGAGCTGCTTC | Amplification fragment containing FRT and Kan^R^ |
| umuD-pKD4_RV | CACCGTCTCACAGCTGGCATAAAACGCGTTTACATCACAGAGGGCAAACAATGGGAATTAGCCATGGTCC | Amplification fragment containing FRT and Kan^R^ |
| dinB-H1_FW | CCAGTGTTGAGAGGTGAGCA | Confirmation of *dinB* deletion |
| dinB-H2_RV | CCCTCATAATAATGCACACCAG | Confirmation of *dinB* deletion |
| polB-H1_FW | CAGGCTATACTCAAGCCTGG | Confirmation of *polB* deletion |
| polB-H2_RV | GCAAAGCATTCGTCACGCATC | Confirmation of *polB* deletion |
| umuD-H1_FW | GATCTGCTGGCAAGAACAGAC | Confirmation of *umuD* deletion |
| umuD-H2_RV | GCGTTTACATCACAGAGGGC | Confirmation of *umuD* deletion |
| lexA.A1_FW | TGAATGGCGAATGGCATTCAAGCCGAATGCTGATTTCCTGCTGCGCGTCAGCGGGATGGCG | Amplification of fragment A |
| lexA.A2_FW | GTCAGCGGGATGGCGATGAAAGATATCGGCATTATG | Amplification of fragment A2 |
| lexA.A2_RV | TTACAGCCAGTCGCCGTTGCGAATA | Amplification of fragment A2 and A |
| lexA.B1_FW | GGCGACTGGCTGTAAGAGCTGCTTCGAAGTTCCTATACT | Amplification of fragment B1 and B |
| lexA.B1_RV | GTTCCTATTCCGAAGTTCC | Amplification of fragment B1 |
| lexA.B2_RV | AATACCGCATCAGGCGATGAAAAACAAACCGCGACGCCAGGCGGCATCGCGGTCTCAGAGATATGGTTCCTATTCCGAAG | Amplification of fragment B |
| pUC19.lexA_FW | ATTCAAGCCGAATGCTGATTTCCTG | Confirmation of the *lexA*(S119A) cassette in pUC19 |
| pUC19.lexA_RV | AAACCGCGACGCCAGGCGGCAT | Confirmation of the *lexA*(S119A) cassette in pUC19 |
| lexA_A_H1KanR_FW | GATCCTTCCTTATTCAAGCCGAATGCTGATTTCCTGCTGCGCGTCAGCGGGATGGCG | Amplification of fragment lexA1 |
| lexA_A_H1KanR_RV | GCCCAGTCATAGCCGAATA | Amplification of fragment lexA1 |
| lexA_B_H2KanR_FW | CTATTCGGCTATGACTGGGCACAACAGACAATCG | Amplification of fragment lexA2 |
| lexA_B_H2KanR_RV | GATGAAAAACAAACCGCGACGCCAGGCGGCATCGCGGTCTCAGAGATATGCATATGAATATCCTCCT | Amplification of fragment lexA2 |
| lexA_FW | GGAATGAAAGCGTTAACGGC | Confirmation of the *lexA*(S119A) cassette in *E. coli* cells |
| lexA600_RV | ACCAGCGTACGGGCTAATGCCT | Confirmation of the *lexA*(S119A) cassette in *E. coli* cells |
| marR-pKD4_FW | CAATATTATCCCCTGCAACTAATTACTTGCCAGGGCAACTAATGTGTAGGCTGGAGCTGCTTC | Amplification fragment containing FRT and Kan^R^ |
| marR-pKD4_RV | GGTAATAGCGTCAGTATTGCGTCTGGACATCGTCATACCTCATGGGAATTAGCCATGGTCC | Amplification fragment containing FRT and Kan^R^ |
| marR170-pKD4_FW | GTTTAAGGTGCTCTGCTCTATCCGCTGCGCGGCGTGTATTACTCGTGTAGGCTGGAGCTGCTTC | Amplification fragment containing FRT and Kan^R^ |
| marR170-pKD4_RV | CTTGGTGCAGGTCCTGGCCAACTAATTGATGGCATTGTATGGGAATTAGCCATGGTCC | Amplification fragment containing FRT and Kan^R^ |
| marR-H1_FW | GCAACTAATTACTTGCCAGGGC | Confirmation of *marR* deletion |
| marR-H2_RV | GTATTGCGTCTGGACATCGT | Confirmation of *marR* deletion |

***Cloning and Purification***

A recombineering protocol adapted from Datsenko and Wanner (1) was used to generate the *ΔdinB*, *ΔpolB*, *ΔumuD*, and triple *ΔdinB ΔpolB ΔumuD* mutants. Briefly, primers were made containing ~50 nucleotides (nt) homologous to the downstream or upstream sequence of the gene we wanted to delete as well as 20 nt of the priming site 1 (pKD4_FW) or 2 (pKD4_RV) of the plasmid pKD4 (for the forward and reverse primer, respectively). These were used to amplify a fragment containing the Kan resistance gene with the FRP sites flanked by a region of homology to the gene of interest. Once the linear substrate was amplified, its sequence was confirmed by Sanger sequencing. The PCR fragment was transformed into *E. coli* MG1655 containing the plasmid pKD46, which has the λ red genes under the control of an arabinose promoter. Colonies that were Kan-resistant were selected and checked for what by PCR using primers H1_FW and H2_RV. For the *ΔdinB*, *ΔpolB*, *ΔumuD* and triple *ΔdinB ΔpolB ΔumuD* mutants, the Kan resistance cassette was removed using FLP recombination (1). Briefly, the cells were transformed with pCP20, then grown overnight at 45˚C to induce FLP expression and selected for the loss of pCP20. Single-candidate recombinants were plated and screened for genomic recombination and plasmid loss.

P1 transduction was used to construct the *ΔdinB ΔpolB umuD* mutant in a step-wise procedure and to move a *lexA*(S119A) cassette into a wild-type MG1655 strain as described previously (2). Briefly, a lysate of the host strain that contained the mutation of interest was made. Then the recipient strain was transduced with the lysate and kanamycin-resistant transductants were checked by PCR.

A *lexA*(S119A) cassette was constructed by cloning four different fragments (A1, A2, B1 and B2) into a pUC19 plasmid. A1 contained 15 bp of pUC19, 50 bp upstream of the *lexA* gene codon 119, and codon 119 with a mutation (T->G in position 355). A2 contained the last 15 bp of A1, and the sequence from codon 119 to the end of the *lexA* open reading frame. B1 contained the last 15 bp of A2 and a kanamycin-resistance cassette flanked by FRT sites. B2 contained the last 15 bp of B1, 50 bp downstream of the *lexA* gene, and 15 bp of pUC19. A2 was obtained by PCR amplifying the chromosome of *E. coli* MG1655 using primers lexA.A2_FW and lexA.A2_RV. B1 was obtained by PCR-amplifying a pKD4 vector using primers lexA.B1_FW and lexA.B1_RV (Supplementary Table 2). A1 and A2 were fused into an A fragment by using primers lexA.A1_FW and lexA.A2_RV. B1 and B2 were fused into a B fragment by using primers lexA.B1_FW and lexA.B2_RV. Fragments A and B were cloned using an In-Fusion kit (In-Fusion® HD Cloning Kit, Takara Bio) into a pUC19 vector linearised with the restriction enzyme SfoI (NEB). All fragments were purified with a PCR clean-up kit (NucleoSpin® Gel and PCR Clean-up, Takara Bio) before being used in any cloning reaction. Chemically competent *E. coli* Stellar cells (Takara Bio) were transformed with the pUC19 vector containing the *lexA*(S119A) cassette as stated by Takara Bio. Colonies that were both ampicillin and kanamycin resistant were selected, grown, used to extract plasmids using a miniprep kit (QIAprep Spin Miniprep Kit, QIAGEN) and checked for the presence of the *lexA*(S119A) cassette by PCR using primers pUC19.lexA_FW and pUC19.lexA_RV. A pUC19 plasmid containing the *lexA*(S119A) cassette was sent for sequencing to confirm the presence of the *lexA* mutation, and then used as a template to amplify the *lexA*(S119A) cassette using primers pUC19.lexA_FW and pUC19.lexA_RV. The *lexA*(S119A) cassette was transformed into electrocompetent *E. coli* MG1655 cells, but all the kanamycin-resistant colonies obtained were false positives. Due to the presence of false positives, the *lexA*(S119A) cassette was split in two fragments (lexA1 and lexA2) using primers lexA_A_H1KanR_FW and lexA_A_H1KanR_RV to amplify lexA1, and primer lexA_B_H2KanR_FW and lexA_B_H2KanR_RV to amplify lexA2. Fragment lexA1 had half of the kanamycin-resistance cassette, and thus, only the cells that incorporated both fragments lexA1 and lexA2 could have the whole kanamycin resistance cassette and be kanamycin resistant. The lexA1 and lexA2 fragments were transformed into electrocompetent *E. coli* EcNR2 cells (3) that have a higher recombination rate. Kanamycin-resistant colonies that had the lexA(S119A) cassette confirmed by PCR using lexA_FW and lexA600_RV primers, were sent for sequencing to confirm the presence of the *lexA*(S119A) cassette. A positive *lexA*(S119A) strain was sent for whole genome sequencing to confirm the genotype.

**Supplementary Results**

Table S3. Minimum Inhibitory Concentrations (MICs) for all antibiotics used against *E. coli* MG1655.

| **Antibiotic** | **Broth-based MIC (µg/mL)** | **Solid-agar MIC (µg/mL)** |
| --- | --- | --- |
| **CIP** | 0.016 | 0.016 |
| **MFX** | 0.032 | ND |
| **NOR** | 0.032 | 0.128 |
| **COU** | 16 | ND |
| **MMC** | 2 | 1.5 |
| **OXO** | 0.4 | 0.4 |
| **CHL** | 4 | 4 |
| **AMP** | 4 | 4 |
| **KAN** | 4 | 6 |
| **TET** | 1.2 | 1.2 |
| **RIF** | ND | 32 |
| **STR** | ND | 24 |
| **TRI** | ND | 0.08 |
| **TMP** | ND | 0.4 |

ND – not done, CIP – ciprofloxacin, MFX – moxifloxacin, NOR – norfloxacin, OXO – oxolinic acid, MMC – mitomycin C, COU – coumermycin A_1_, AMP – ampicillin, KAN – kanamycin, CAM – chloramphenicol, TET – tetracycline, RIF – rifampin, STR – streptomycin, TRI – triclosan, TMP – trimethoprim.

Table S4. Broth-based Minimum Inhibitory Concentrations (MICs) for ciprofloxacin (CIP) against all *E. coli* strains used.

| ***E. coli* Strain** | **MIC (µg/mL)** |
| --- | --- |
| MG1655 wild type | 0.016 |
| MLS83L | 0.256 |
| MG1655 ∆*recA* | 0.006 |
| MG1655 *lexA*(S119A) | 0.008 |
| MG1655 ∆*dinB* | 0.032 |
| MG1655 ∆*polB* | 0.032 |
| MG1655 ∆*umuD* | 0.021 |
| MG1655 Δ*dinB* Δ*polB* Δ*umuD* | 0.032 |

**Table S5. All variants and mutations identified from whole genome sequencing of *E. coli* MG1655 strains from QIAR.**

(See attached Excel spreadsheet.)

**Figure S1**


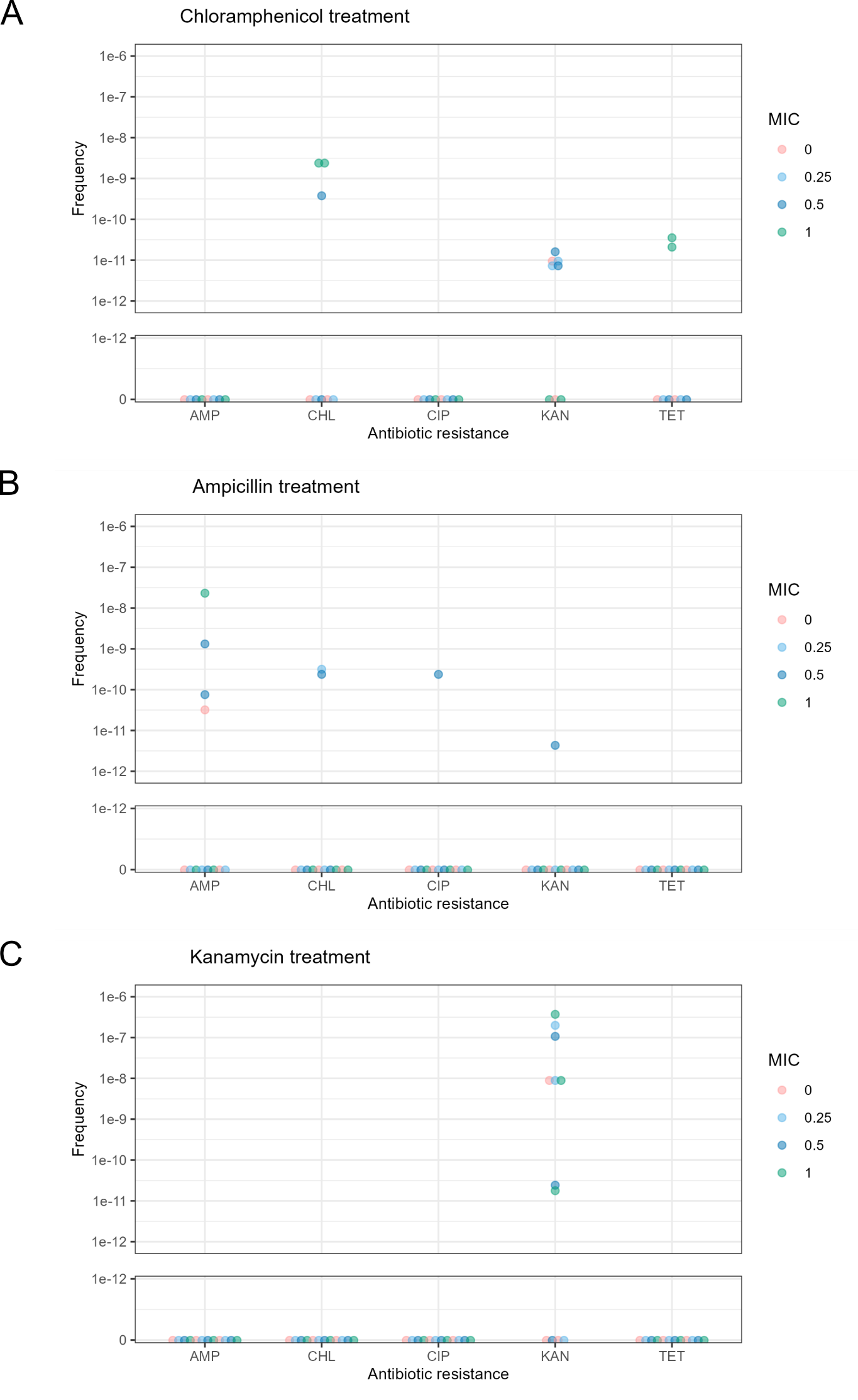


FIG S1. Frequency of antibiotic resistance per CFU for *E. coli* MG1655 treated with sublethal levels of a) chloramphenicol, b) ampicillin or c) kanamycin. Each dot represents a data point at 0, 0.25, 0.5 or 1× the MICs of the respective antibiotics. The X axis shows the antibiotics to which resistance was tested for: CHL = chloramphenicol, TET= tetracycline, CIP = ciprofloxacin. The Y axis represents the frequency of resistant colonies per CFU split into a linear scale from 0 to 1e-12 and a logarithmic scale from 1e-12 to 1e-6.

Figure S2


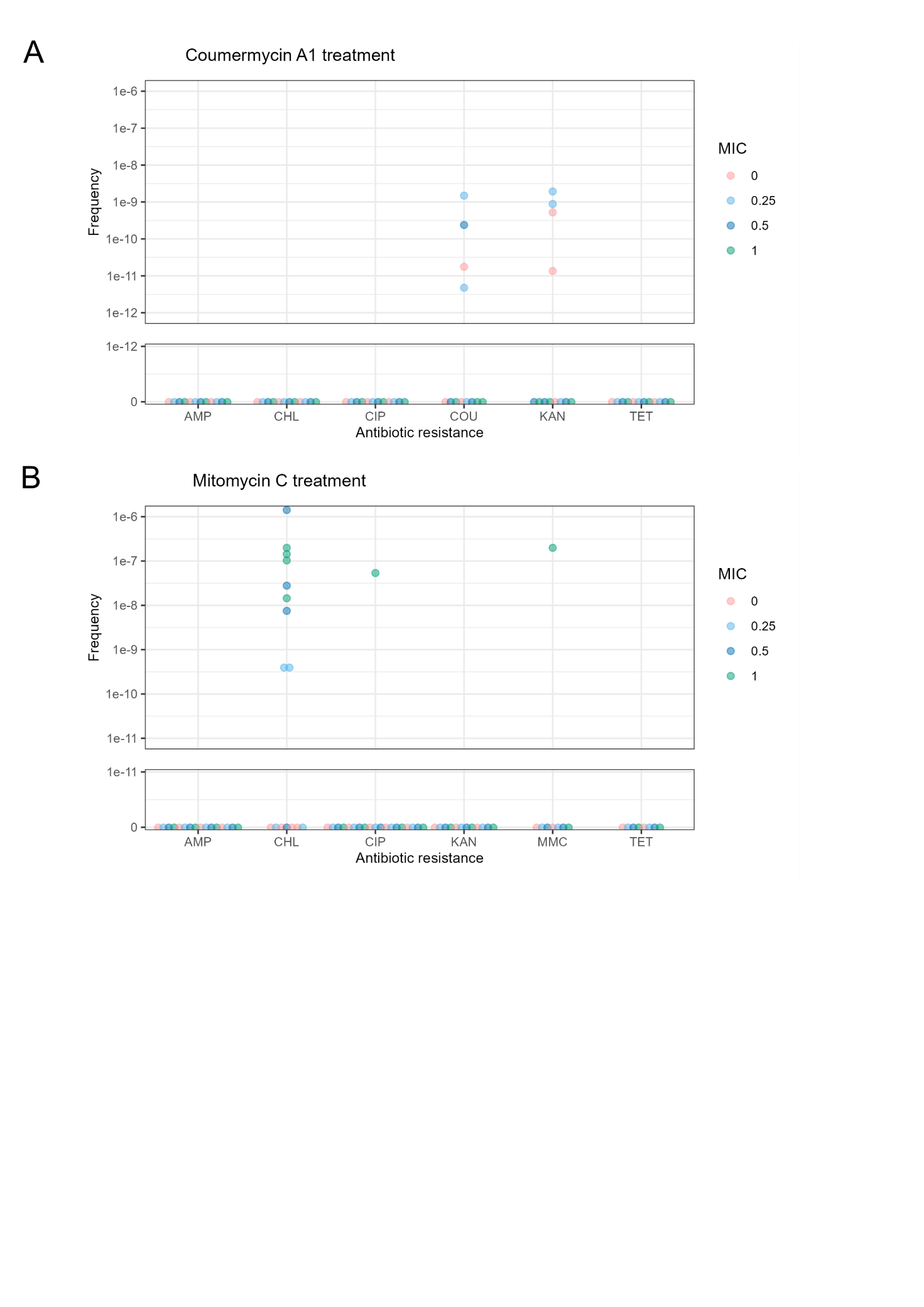


Figure S2. Frequency of antibiotic resistance per CFU for *E. coli* MG1655 treated with sublethal a) coumermycin A1 (COU) or b) mitomycin (MMC) over a 24 h incubation. Each dot represents a data point at 0, 0.25, 0.5 or 1× the MICs of the respective antibiotics. The X axis shows the antibiotics to which resistance was tested for: KAN = kanamycin, CHL = chloramphenicol, TET = tetracycline, AMP = ampicillin, CIP = ciprofloxacin. The Y axis represents the frequency of resistant colonies per CFU split into a linear scale from 0 to 1e-11 and a logarithmic scale from 1e-11 to 1e-6.

**Figure S3**


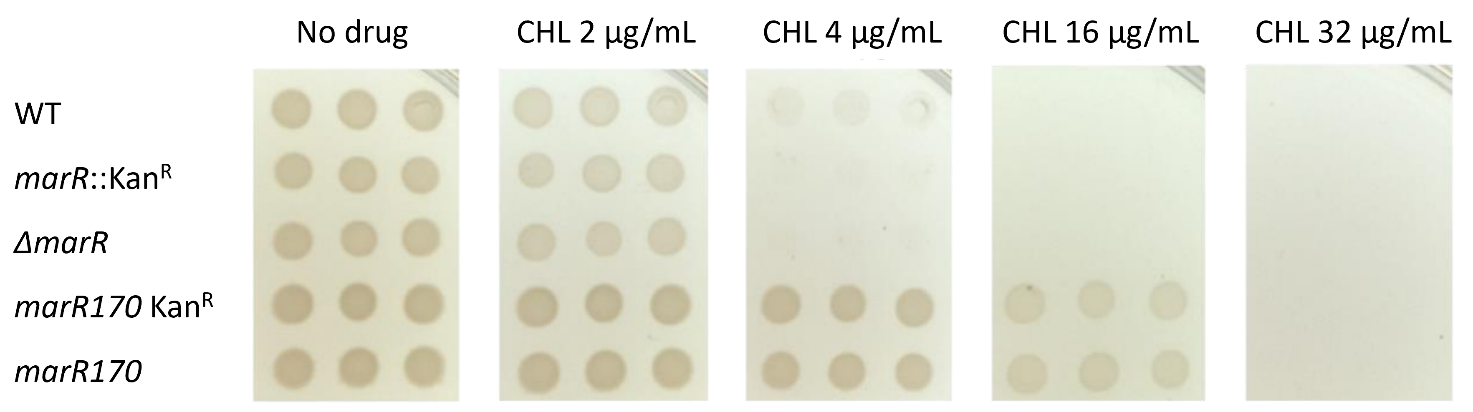


Figure S3. Chloramphenicol (CHL) MIC of *marR* mutants. *mar170* is a truncated version of *marR* that has a 157 base pair deletion starting from base pair 170.

**Supplementary References**

1. Datsenko KA, Wanner BL. 2000. One-step inactivation of chromosomal genes in Escherichia coli K-12 using PCR products. Proc Natl Acad Sci U S A 97:6640-5.

2. Thomason LC, Costantino N, Court DL. 2007. E. coli Genome Manipulation by P1 Transduction. Current Protocols in Molecular Biology 79:1.17.1-1.17.8.

3. Ryu YS, Biswas RK, Shin K, Parisutham V, Kim SM, Lee SK. 2014. A simple and effective method for construction of Escherichia coli strains proficient for genome engineering. PLoS One 9:e94266.
